# Decrease of miR-195 Promotes Chondrocytes Proliferation and Maintenance of Chondrogenic Phenotype via Targeting FGF-18 Pathway

**DOI:** 10.3390/ijms18050975

**Published:** 2017-05-04

**Authors:** Yong Wang, Tao Yang, Yadong Liu, Wei Zhao, Zhen Zhang, Ming Lu, Weiguo Zhang

**Affiliations:** 1 Department of Joint Surgery, The First Affiliated Hospital of Dalian Medical University, Dalian 116011, China; WY_landy1116@163.com (Y.W.); yangtao_07@126.com (T.Y.); doctoryadong@163.com (Y.L.); doctorzz@126.com (Z.Z.); dllm@2008.sina.com (M.L.); 2 The 4th Department of Orthopedic Surgery, The Central Hospital Affiliated to Shenyang Medical College, Shenyang 110024, China; zhaowei332@126.com

**Keywords:** miR-195, FGF18, cartilage lesion, chondrocyte proliferation, Col2a1/aggrecan, signal pathway

## Abstract

Slow growth and rapid loss of chondrogenic phenotypes are the major problems affecting chronic cartilage lesions. The role of microRNA-195 (miR-195) and its detailed working mechanism in the fore-mentioned process remains unknown. Fibroblastic growth factor 18 (FGF-18) plays a key role in cartilage homeostasis; whether miR-195 could regulate FGF-18 and its downstream signal pathway in chondrocyte proliferation and maintenance of chondrogenic phenotypes still remains unclear. The present research shows elevated miR-195 but depressed FGF-18 expressed in joint fluid specimens of 20 patients with chronic cartilage lesions and in CH1M and CH3M chondrocytes when compared with that in joint fluid specimens without cartilage lesions and in CH1W and CH2W chondrocytes, respectively. The following loss of function test revealed that downregulation of miR-195 by transfection of miR-195 inhibitors promoted chondrocyte proliferation and expression of a type II collagen α I chain (Col2a1)/aggrecan. Through the online informatics analysis we theoretically predicted that miR-195 could bind to a FGF-18 3′ untranslated region (3′UTR), also, we verified that a miR-195 could regulate the FGF-18 and its downstream pathway. The constructed dual luciferase assay further confirmed that FGF-18 was a direct target of miR-195. The executed anti-sense experiment displayed that miR-195 could regulate chondrocyte proliferation and Col2a1/aggrecan expression via the FGF-18 pathway. Finally, through an in vivo anterior cruciate ligament transection (ACLT) model, downregulation of miR-195 presented a significantly protective effect on chronic cartilage lesions. Evaluating all of the outcomes of the current research revealed that a decrease of miR-195 protected chronic cartilage lesions by promoting chondrocyte proliferation and maintenance of chondrogenic phenotypes via the targeting of the FGF-18 pathway and that the miR-195/FGF-18 axis could be a potential target in the treatment of cartilage lesions.

## 1. Introduction

It is common to see cartilage lesions caused by external factors such as sports injuries, trauma, unstable joints, and degenerative arthritis, all of which may be frequently experienced in modern society [1,2]. Mature chondrocytes are slow-metabolic, limited-proliferated, highly-differentiated cells, and the self-repairing ability of cartilage is confined [3,4]. Also, the repair of cartilage lesions continues to be an urgent concern for clinical doctors in sports medicine and joint surgery. For the past few years, methods including autologous chondrocyte implantation (ACI), autogenic or allogenic cartilage transplantation, tissue-engineered cartilage transplantation and microfracture techniques have been widely used for treatment of chronic cartilage lesions and have achieved a certain clinical effect, but the long-term effect remains unsatisfactory [5,6,7,8,9,10,11,12]. Presently, ACI is mainly used for more than 2.5–3 cm^2^ full-thickness cartilage defects and certain therapeutic effects are acquired, but some problems such as an insufficiency of autologous donor cells and the difficulties of the in vitro process to swiftly and reliably amplify the chondrocyte remains unresolved [13,14,15,16]. The major problems of in vitro amplification of chondrocytes are its poor proliferation ability and the fast dedifferentiation phenomenon (i.e., loss of chondrogenic phenotypes). Hence, finding a proper target gene to promote chondrocytes proliferation and to maintain the chondrogenic phenotypes during in vitro procedures is particularly important.

MicroRNAs (miRNAs), 22–25 nucleotides in length, are a group of small non-coding RNAs which are involved in many diseases that include cartilage lesions and their repair [17,18,19]. MicroRNA-195 (miR-195) is widely reported in numerous malignant tumors such as osteosarcoma, breast cancer, colorectal cancer, prostate cancer, and non-small lung cancer [20,21,22,23,24]. Through an early clinical detection trial we found that miR-195 was elevated in joint fluid specimens of patients with chronic cartilage lesions, so we wondered whether miR-195 was involved in the process of cartilage lesion formation and its repair. It was reported that miR-195 could inhibit the chondrocytes proliferation by targeting the regulation of G-protein coupled receptor kinase interacting protein-1 (GIT1) [25]. Bai et al. found that miR-195 could target hypoxia-inducible factor 1 α (HIF-1α) and promote apoptosis in hypoxic chondrocytes [26]. However, related reports that consider whether miRNAs could regulate chondrogenic phenotype maintenance or affect the chondrocytes dedifferentiation are even rarer. Seidl and colleagues reported that inhibition of miR-138 contributed to the maintenance of the chondrocyte phenotype and depressed the dedifferentiation progression in cultured human articular chondrocytes [27]. Hong and colleagues reported that miR-221/-222 was increased during the chondrocytes dedifferentiation [28]. Presently, whether miR-195 could affect chondrocyte proliferation and maintenance of chondrogenic phenotypes remains unclear.

Fibroblast growth factor (FGF) phenomena are in evidence with regard to cell growth, differentiation, angiogenesis, inflammation, and other developmental processes such as skeletal development [29,30]. As a representative of the FGF family, fibroblastic growth factor 18 (FGF-18) plays a key role in skeletal development. [31]. Recent studies showed that FGF-18 was mainly distributed in perichondrium and joint space locations to work as a promoter in chondrocyte proliferation [32]. Bradley and colleagues reported that the inhibition of PH-domain and leucine-rich repeat protein phosphatase 1 (Phlpp1) promoted chondrocyte proliferation via the upregulation of FGF-18 expression [33]. Davidson et al. found that FGF-18 stimulated chondrocyte proliferation and matrix production via regulation of its downstream fibroblastic growth factor receptor 3 (FGFR3) [34]. It was reported that intra-articular injection of FGF-18 presented a protective effect on cartilage lesions [35,36]. To date, whether any miRNA could regulate FGF-18 to influence chondrocyte proliferation and chondrogenic phenotype maintenance remains unclear.

In the present study, we revealed that miR-195 could target FGF-18 and regulate the activity of FGF-18 downstream and that inhibition of miR-195 protects chronic cartilage lesion formation by promoting chondrocyte proliferation and maintenance of the chondrogenic phenotype via the targeting of the FGF-18 pathway.

## 2. Results

### 2.1. Expression of miR-195 and FGF-18 in the Joint Fluid of Patients with Chronic Cartilage Lesions and with Respect to the Different Ages of Chondrocytes

**Figure 1:**
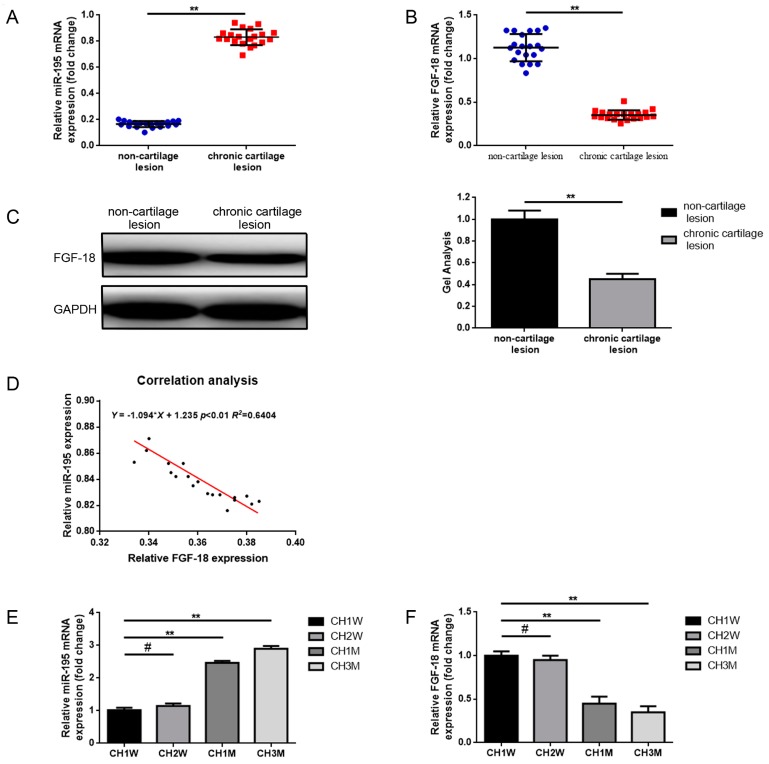

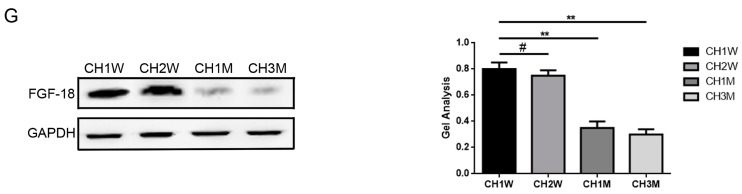
Expression of microRNA-195 (miR-195) and fibroblast growth factor 18 (FGF-18) in joint fluid of patients with chronic cartilage lesion and in different ages of chondrocytes. (**A**–**C**) Elevated miR-195 (**A**) but decreased FGF-18 (**B**,**C**) expression in joint fluid specimens of 20 patients with chronic cartilage lesions and in paired specimens of 20 patients with non-cartilage lesions were determined by real-time PCR and western blot test methods using glyceraldehyde-3-phosphate dehydrogenase (GAPDH) as an internal control. The mRNA and protein expression levels were normalized to non-cartilage lesion groups. ** *p* < 0.01 vs. non-cartilage lesion groups; (**D**) a significant negative correlation was revealed by a two-tailed Pearson’s correlation analysis, *r* = 0.6404, *p* < 0.01. (**E**–**G**) Chondrocytes isolated from 1-week-old, 2-week-old, 1-month-old, and 3-month-old Sprague-Dawley female rats were respectively named as CH1W, CH2W, CH1M, and CH3M. The expression of miR-195 and FGF-18 was also detected in the above-mentioned four groups of chondrocytes, and an upregulated miR-195 but downregulated FGF-18 expressions were displayed by real-time PCR and western blot test methods using GAPDH as an internal control. The mRNA and protein expression levels were normalized to the CH1W group. ** *p* < 0.01 vs. CH1W group. # *p* > 0.05 vs. CH1W group. All experiments were repeated in triplicate and all data was shown as a mean ± standard deviation (S.D.) (*n* = 3, each).

miR-195 expression was measured by real-time PCR while FGF-18 expression was detected by the use of real-time PCR and western blot test methods to evaluate joint fluid specimens of 20 patients with chronic cartilage lesions and in paired specimens of 20 patients with non-cartilage lesions. As shown in Figure 1A, miR-195 expression was noticeably elevated in the chronic cartilage lesion group compared to the non-cartilage lesion group (*p* < 0.01). By contrast, the expression of FGF-18 was noticeably decreased in the chronic cartilage lesion group when compared to the non-cartilage lesion group (Figure 1B,C) (*p* < 0.01). Meanwhile, the relationship between miR-195 and FGF-18 expression was analyzed by using a two-tailed Pearson’s correlation analysis, as revealed in Figure 1D, a negative correlation between the expression of miR-21 and FGF-18 was identified (*p* < 0.01).

It is well known that compared to premature chondrocytes, the main problems observed during in vitro expansion of mature chondrocytes are poor proliferation ability and the phenomenon of dedifferentiation [37,38]. Since miR-195 and FGF-18 are involved in chondrocyte proliferation and dedifferentiation, as previously reported [25,32,39], we wondered whether both of them are differentially expressed with respect to the different ages of chondrocytes. We isolated different ages of chondrocytes from 1-week-old, 2-week-old, 1-month-old, and 3-month-old rats (named as CH1W, CH2W, CH1M, and CH3M, respectively) and checked the expression of miR-195 and FGF-18 in the formerly mentioned chondrocytes. According to the results of real-time PCR, the expression of miR-195 was gradually elevated in chondrocytes with regard to aging (Figure 1E) (*p* < 0.01). Correspondingly, the FGF-18 expression was gradually decreased in chondrocytes with regard to aging (Figure 1F,G) (*p* < 0.01).

### 2.2. Decrease of miR-195 Promotes Chondrocytes Proliferation and Col2a1/Aggrecan Expression

**Figure 2:**
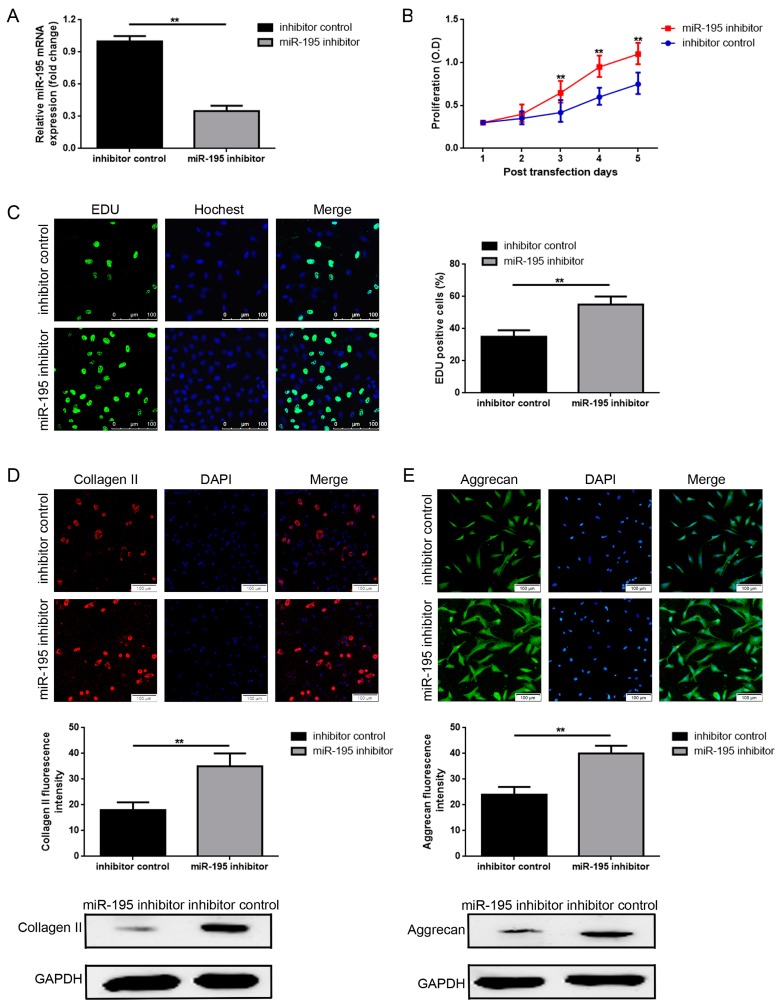
Decrease of miR-195 promotes chondrocyte proliferation and Col2a1/aggrecan expression. (**A**) miR-195 expression was remarkably depressed by transfection of the miR-195 inhibitor when normalized to the inhibitor control group, as measured by real-time PCR using the internal control of GAPDH. ** *p* < 0.01 vs. an inhibitor control group. (**B**,**C**) Suppression of miR-195 inhibited chondrocyte proliferation as determined by a Cell Counting Kit-8 (CCK-8) assay (**B**) and 5-Ethynyl-20-Deoxyuridine (EDU) (**C**). (**D**,**E**) Inhibition of miR-195 suppressed Col2a1 (**D**) and aggrecan (**E**) expression in chondrocytes checked by immunofluorescence staining and western blot test methods. (Magnification, 200×; scale bar, 100 μm). ** *p* < 0.01 vs. inhibitor control group. All experiments were repeated in triplicate and all data were shown as a mean ± S.D. (*n* = 3, each).

Since miR-195 was upregulated in chronic cartilage lesion patients and in chondrocytes with aging, we constructed the loss-of-function experiment to elucidate how the function of miR-195 might work in chondrocytes with special emphasis on proliferation and maintenance of chondrogenic phenotypes—expression of Col2a1 and aggrecan. First of all, passage 2 of CH3M chondrocytes were selected for all the following cellular detections. Secondly, as shown in Figure 2A, transfection of a miR-195 inhibitor into chondrocytes resulted in significant depression of miR-195 expression when compared to inhibitor control (*p* < 0.01). The outcomes of Cell Counting Kit-8 (CCK-8) and 5-Ethynyl-20-Deoxyuridine (EDU) indicated that the depression of miR-195 (transfection of miR-195 inhibitor) promoted chondrocyte proliferation (Figure 2B,C) (*p* < 0.01). Meanwhile, those observed with immunofluorescence staining, inhibition of miR-195 also promoted Col2a1 and aggrecan expression in chondrocytes (Figure 2D,E) (*p* < 0.01).

### 2.3. miR-195 Targets FGF-18 and Regulates Its Downstream Pathway

**Figure 3:**
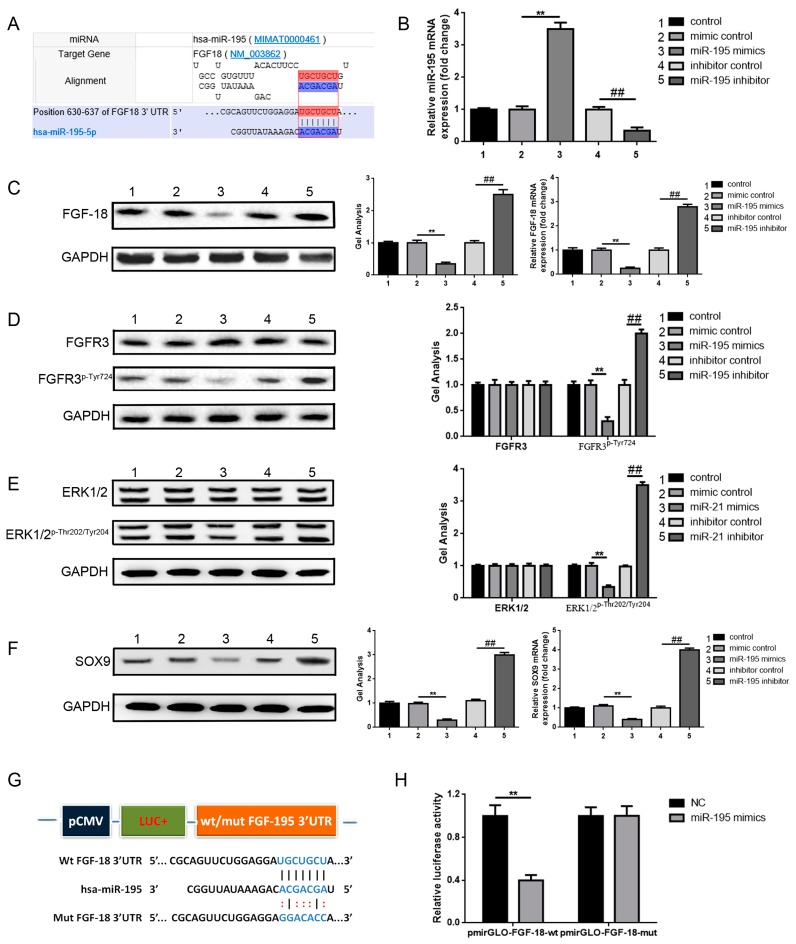
miR-195 targets FGF-18 and regulates its downstream pathway. (**A**) FGF-18 3′UTR had a binding site containing seven bases for miR-195, as predicted by online prediction software TargetScan (Available online: http://www.targetscan.org) and RNAhybrid (Available online: https://bibiserv.cebitec.uni-bielefeld.de/rnahybrid); (**B**) miR-195 expression after different intervention when normalized to the control group was detected by real-time PCR and GAPDH and was set as an internal control. (**C**–**F**) miR-195 could regulate FGF-18 expression (**C**), then affect the phosphorylation level of FGFR3 (FGFR3^p-Tyr724^) (**D**), ERK1/2 (ERK1/2^p-Thr202/Tyr204^) (**E**), and affect the expression level of SOX (**F**) as determined by western blot test method and real-time PCR. GAPDH was set as the internal control and all data were normalized to the control group, respectively. ** *p* < 0.01 vs. mimic control group, ## *p* < 0.01 vs. inhibitor control group. (**G**) Diagram of the luciferase reporter plasmids with the wild-type or mutant FGF-18 3′UTR. (**H**) Relative luciferase activity was achieved through a dual luciferase reporter assay. ** *p* < 0.01 vs. NC + pmirGLO-FGF-18-wt group. All experiments were repeated in triplicate and all data were shown as a mean ± S.D. (*n* = 3, each).

According to the above outcomes, we verified that miR-195 was involved in chondrocyte proliferation and the maintenance of chondrogenic phenotypes and that FGF-18 was ectopic and expressed in joint fluid specimens of patients with and without chronic cartilage lesions and in the different aging of chondrocytes. Further, we wondered about the potential relationship between miR-195 and FGF-18. First, we theoretically predicted that miR-195 could combine with the FGF-18 3′untranslated region (3′UTR) through the use of online predictive software TargetScan (Available online: http://www.targetscan.org) and a RNAhybrid (Available online: https://bibiserv.cebitec.uni-bielefeld.de/rnahybrid) (Figure 3A). Second, we confirmed that up- and down-regulation of miR-195 could negatively affect FGF-18 and the phosphorylation level of FGFR3 (FGFR3^p-Tyr724^)—a downstream receptor of the FGF-18 signal pathway—to a significant level (3B-D). Third, as the results show in Figure 3E,F, miR-195 regulated the phosphorylation level of ERK1/2 (ERK1/2^p-Thr202/Tyr204^), a proliferation-related downstream target of FGF-18, and the expression of SOX9, a chondrogenic phenotype that is maintenance-related downstream of FGF-18. Finally, a dual luciferase reporter assay was conducted to finally confirm the target binding effect between miR-195 and FGF-18 3′UTR. We constructed a luciferase reporter of plasmids pmirGLO-FGF-18-wt and pmirGLO-FGF-18-mut that respectively contained a wild type and mutant FGF-18 3′UTR (Figure 3G). The constructed luciferase reporter plasmids and miR-195 mimics were co-transfected into HEK-293 cells (miR-195 mimic control was set as negative control, NC) and the fluorescence changes were observed to evaluate the potential target binding effect. As the results showed in Figure 3H, compared to NC, co-transfection of miR-195 mimics and pmirGLO-FGF-18-wt resulted in significant weakness of fluorescence, but the weakened effect vanished when the theoretical miR-195 binding sites in FGF-18 was mutated (co-transfection of miR-195 mimics and pmirGLO-FGF-18-mut). These outcomes verified that miR-195 could target FGF-18 and regulate its downstream pathway.

### 2.4. FGF-18 Rescues the Inhibitive Effect of miR-195 on Chondrocyte Proliferation and Col2a1/Aggrecan Expression

**Figure 4:**
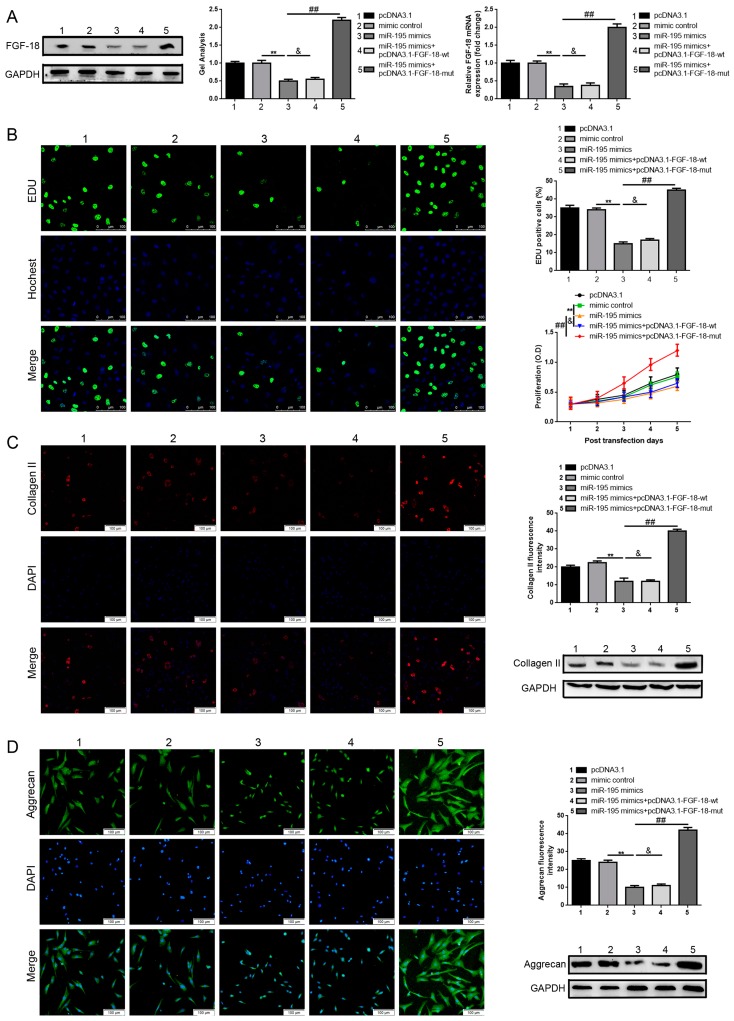
FGF-18 rescues the inhibitive effect of miR-195 on chondrocyte proliferation and Col2a1/aggrecan expression. (**A**) miR-195 mimics prominently depressed FGF-18 expression and the depressive effect was rescued by pmirGLO-FGF-18-mut, as detected by western blot and real-time PCR; (**B**) miR-195 mimics significantly inhibited chondrocyte proliferation and the inhibitive effect was reversed by pmirGLO-FGF-18-mut, as measured by CCK-8 assay and EDU; (**C**,**D**) MiR-195 mimics crippled Col2a1 (**C**) and aggrecan (**D**) expression and the crippled effect was rescued by pmirGLO-FGF-18-mut and evaluated by immunofluorescence staining. (Magnification, 200×; scale bar, 100 μm). ** *p* < 0.01 vs. miR-195 mimics group. ## *p* < 0.01 vs. miR-195 mimics group. & *p* > 0.05 vs. miR-195 mimics group. All experiments were repeated in triplicate while all data were normalized to pcDNA3.1 group and were shown as a mean ± S.D. (*n* = 3, each).

In the above results section, we found that suppression of miR-195 could promote chondrocyte proliferation and maintenance of chondrogenic phenotypes, furthermore, we verified that miR-195 targeted FGF-18 and regulated it downstream. It was reported that the FGF-18/FGFR3 pathway played a key role in chondrocyte proliferation and Col2a1 and proteoglycan expression [32,34,40]. Hence, we designed an anti-sense experiment to verify whether the inhibitive effect miR-195 played on chondrocyte proliferation and maintenance of chondrogenic phenotypes was achieved through the FGF-18 pathway. First, we constructed the FGF-18 over-expression plasmids pcDNA3.1-FGF-18-wt and pcDNA3.1-FGF-18-mut that respectively contained wild type and mutant miR-195 binding sites. Second, the results of western blot test methods and real-time PCR revealed that transfection of miR-195 mimics could predictably inhibit FGF-18 expression, but that the inhibitive effect could be reversed by pcDNA3.1-FGF-18-mut but not by pcDNA3.1-FGF-18-wt (Figure 4A). Also, we re-executed the EDU, CCK-8, and immunofluorescence staining to check the proliferation ability and the expression level changes of Col2a1/aggrecan in chondrocytes. As can be observed in the results presented in Figure 4B–D, compared to pcDNA3.1 and mimic control, transfection of miR-195 mimics were shown to remarkably decrease both the ability to proliferate and the expression level of Col2a1/aggrecan in chondrocytes. However, the inhibitive effect was rescued by pcDNA3.1-FGF-18-mut (co-transfection of miR-195 mimics and pcDNA3.1-FGF-18-mut) but not by pcDNA3.1-FGF-18-wt (co-transfection of miR-195 mimics and pcDNA3.1-FGF-18-wt). Taking all the aforementioned outcomes together indicated that miR-195 could inhibit chondrocyte proliferation and maintenance of chondrogenic phenotypes through the FGF-18/FGFR3 pathway.

### 2.5. Downregulation of miR-195 Protects Chronic Cartilage Lesions In Vivo

**Figure 5:**
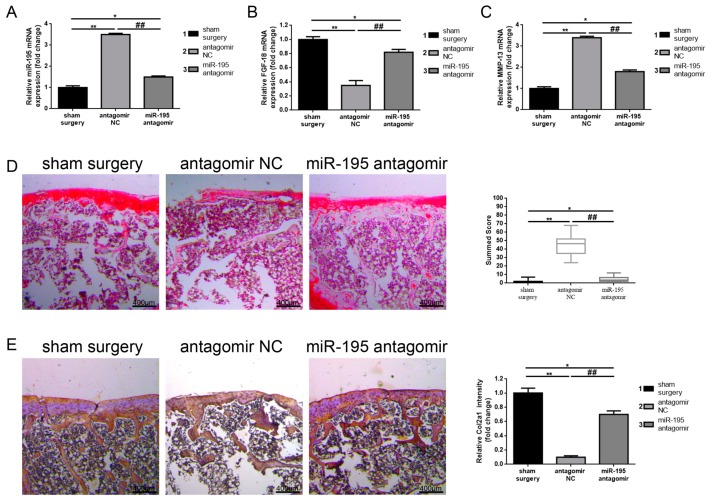

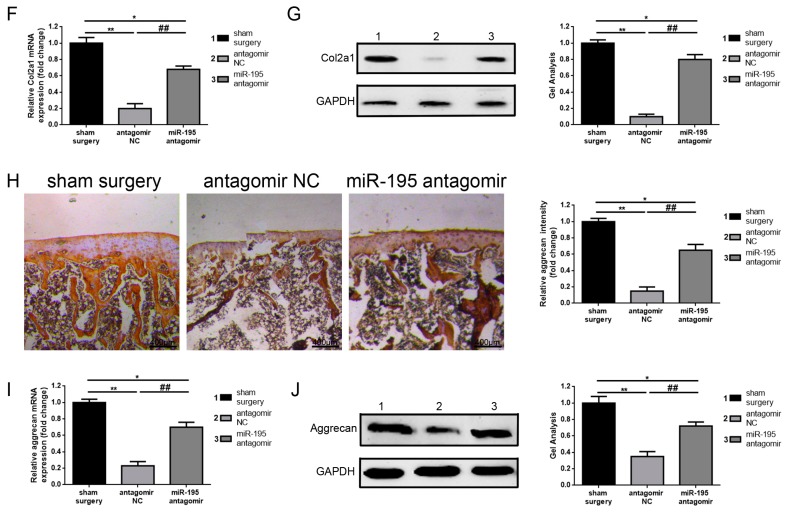
Downregulation of miR-195 protects chronic cartilage lesion in vivo. (**A**–**C**) Anterior cruciate ligament transection (ACLT) led to elevated miR-195 (**A**) and matrix metalloprotease-13 (MMP-13) (**C**) but depressed FGF-18 (**B**) mRNA expression, but the increased or decreased effects were reversed by miR-195 antagomir, as determined by real-time PCR. (**D**) Safranin O staining revealed significant cartilage lesion by ACLT in antagomir NC group but an obvious protective effect in the miR-195 antagomir group (depression of miR-195). (**E**–**G**) Immunohistochemistry (IHC) (**E**), real-time PCR (**F**) and western blot (**G**) presented a remarkable decrease of Col2a1 (**E**) by ACLT in the antagomir NC group, while the weakening effect was reversed in the miR-195 antagomir group (depression of miR-195). (**H**–**J**) Immunohistochemistry (IHC) (**H**), real-time PCR (**I**) and western blot (**J**) presented a remarkable decrease of aggrecan by ACLT in the antagomir NC group, while the weakening effect was reversed in the miR-195 antagomir group (depression of miR-195). (Magnification, 40×; scale bar, 400 μm). * *p* < 0.05, ** *p* < 0.01 vs. sham surgery group. ## *p* < 0.01 vs. antagomir NC group. All experiments were repeated in triplicate while all data were normalized to the sham surgery group and were shown as mean ± S.D. (*n* = 3, each).

To further explore the functional role that miR-195 may play with regard to chronic cartilage lesion repair in vivo, we constructed anterior cruciate ligament transection (ACLT) models as previously reported to simulate the process of chronic cartilage lesion formation. Meanwhile, a sterile saline, antagomir negative control (antagomir NC) and miR-195 antagomir were injected into joint cavities in each group, respectively. The expression of matrix metalloprotease-13 (MMP-13) and Safranin O staining were used to evaluate the degree of chronic cartilage lesion formation. As can be observed in the results displayed in Figure 3A, compared to a sham surgery group, ACLT (injection of antagomir NC) resulted in the significant elevation of miR-195, but the promoted effect was inhibited by suppression of miR-195 (injection of miR-195 antagomir). Furthermore, the expression of FGF-18 presented a contrary tendency compared with miR-195. Also, we measured the expression of MMP-13—a marker of catabolism of cartilage—to access the role that miR-195 might have in cartilage degradation. As presented in Figure 5C, compared to the sham surgery group, ACLT led to a high-expression of MMP-13 which indicated an acceleration of cartilage degradation, but the catabolic effect was suppressed by inhibition of miR-195. Additionally, Safranin O staining tests revealed lesser staining, even a rough articular surface and significant high semi-quantitative grading score in the ACLT group when compared to the sham surgery group, but the phenomenon was reversed by the downregulation of miR-195 (injection of miR-195 antagomir) (Figure 5D). The aforementioned results indicated that downregulation of miR-195 had a protective effect on chronic cartilage lesions. In addition, the expression of Col2a1/aggrecan in each group was also detected by immunohistochemistry (IHC), real-time PCR, and western blot. As shown in Figure 5E–G, compared to the sham surgery group, ACLT caused a prominent depression of Col2a1 expression, but the effect was reversed by the decrease of miR-195. Finally, the findings of an aggrecan detection displayed the same tendency as Col2a1 (Figure 5H–J). Summarily, the outcomes revealed that downregulation of miR-195 promoted Col2a1/aggrecan expression and had a protective on chronic cartilage lesions.

## 3. Discussion

Cartilage repair is a disturbing problem for clinical doctors because of the slow-metabolic, highly differentiated characteristics, and limited self-repair capacity of mature chondrocytes [41,42,43]. Though surgical therapeutic methods for cartilage repair achieved certain encouraging outcomes, gene therapy based strategies in the repair of cartilage lesions remain a hotspot in basic cartilage-related research [44,45]. miRNAs and their target genes of mediated regulation of cartilage development, hemostasis, and regeneration provide new insight for post-damage repair of cartilage [19,46,47].

miR-195 is located at chromosome 17p13.1 and has been widely characterized as a tumor suppressor gene in various cancers including prostate cancer, breast cancer, hepatocellular carcinoma, osteosarcoma, and others [48,49,50,51]. Also, miR-195 was reported to take part in aortic aneurysmal disease, mesenchymal stem cells proliferation and osteogenesis, Hirschsprung’s disease, and chondrocyte hemostasis [26,52,53,54]. Almeida et al. reported that overexpression of miR-195 led to a decrease in osteogenic differentiation and proliferation rate in human primary mesenchymal stromal/stem cells (MSC) [55]. Zhou and colleagues reported that miR-195 suppressed cervical cancer migration and invasion through targeting Smad3 [56]. In the present study, we found that miR-195 was elevated in joint fluid specimens of patients with chronic cartilage lesions and in CH1M/CH3M chondrocytes. It is well known that miRNAs could regulate their downstream genes via binding to the 3′UTR of the targeted genes [57,58,59]. Here, through an online bioinformatics analysis and the executed dual luciferase assay, we verified that miR-195 could target and bind to a FGF-18 3′UTR and regulate FGF-18 mRNA and protein expression, and further affect the phosphorylation level of FGFR3—a downstream receptor of FGF-18. As to the functional experiment, we revealed that miR-195 played a key role in chondrocyte proliferation and the maintenance of chondrogenic phenotypes.

**Figure 6:**
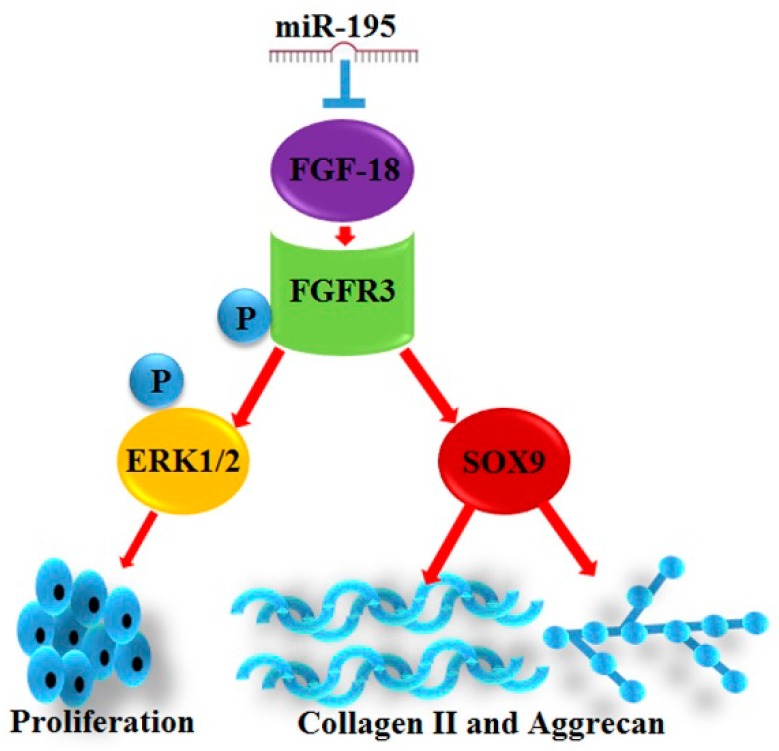
Schematic diagram of mechanism of this research.

FGF-18 is a novel growth factor first reported in 1998, and belongs to the FGF family, which are comprised of 23 members [31]. It was reported that FGF-18 could bind to FGFR3 with a high affinity and played a key role in chondrocyte proliferation [60,61]. In the current study, we confirmed that FGF-18 was decreased in chondrocytes with age as a factor (CH1M and CH3M) and in joint fluid specimens of patients with chronic cartilage lesions. Meanwhile, we verified that upregulation of miR-195 suppressed FGF-18 expression and the activity level of its downstream FGFR3/ FGFR3^p-Tyr724^, ERK1/2/ERK1/2^p-Thr202/Tyr204^, and SOX9. SOX9, which was well accepted as the upstream regulator of Col2a1 and aggrecan [62,63,64,65,66], was reported as being regulated by FGF-18 according to former studies [67,68]. Furthermore, in the present study, we also showed that ectopic miR-195 could correspondingly regulate the expression of FGF-18 and its downstream SOX9 and further effect the expression of Col2a1 and aggrecan. Simultaneously, we revealed that miR-195 could regulate the phosphorylation level of ERK1/2—an acknowledged proliferation-related gene. Through the antisense experiment, we showed that the elevation of miR-195 by transfection of miR-195 mimics resulted in significant inhibition of chondrocyte proliferation and Col2a1/aggrecan expression. However, the inhibitive effect was reversed by the FGF-18 cDNA plasmids containing mutant (pcDNA3.1-FGF-19-mut) but wild (pcDNA3.1-FGF-19-wt) type binding sites for miR-195. This phenomenon strongly confirmed that miR-195 could regulate chondrocyte proliferation and maintenance of chondrogenic phenotypes through a FGF-18 pathway (Figure 6). Finally, through the in vivo ACLT model, we confirmed that depression of miR-195 promoted Col2a1 and aggrecan expression and presented a protective effect on chronic cartilage lesions.

In conclusion, as the schematic diagram displayed in Figure 6 illustrates, the present research reveals that FGF-18 was a target of miR-195 and that miR-195 could negatively regulate FGF-18 expression and affect chondrocyte proliferation via the ERK1/2 pathway and affect maintenance of chondrogenic phenotypes via the SOX9 pathway. Meanwhile, the current study might provide a new regulating target in chondrocyte amplification in vitro. Also, the present study indicated that miR-195/FGF-18 axial might be a new target in cartilage repair.

## 4. Materials and Methods

### 4.1. Patients and Tissue Samples

The joint fluid specimens of 20 patients with chronic cartilage lesions and in paired specimens of 20 patients with non-cartilage lesions, as verified by arthroscopy according to the International Cartilage Repair Society (ICRS) classification system, were collected in the first affiliated hospital of Dalian Medical University. The ICRS sore of the patients with chronic cartilage lesions were grade III in 12 cases and grade IV in 8. All the samples were obtained at the time of surgery and immediately sent to the central laboratory of Dalian Medical University for further testing. The trial was approved by the Institute Research Medical Ethics Committee of Dalian Medical University (16 May 2016, No. 2016MAR22-13). Written informed consent was obtained from all participants.

### 4.2. Cell Isolation and Culture 

Primary chondrocytes were isolated as previously described from 1-week-old, 2-week-old, 1-month-old, and 3-month-old Sprague-Dawley female rats and named as CH1W, CH2W, CH1M, and CH3M, respectively [69,70]. In brief, the cartilage from the femoral head, femoral condyles, and tibia plateau were stripped, diced, and digested twice in 3 mg/mL collagenase (Gibco, El Paso, TX, USA) for 1 h and then 0.5 mg/mL overnight at 37 °C. Undigested cartilage was filtered out and the remaining filtrate containing chondrocytes was centrifuged for 5 min, 1000 rpm. Next, the supernatant was discarded and the pellet was re-suspended in Dulbecco’s Modified Eagle Media: Nutrient Mixture F-12 (DMEM/F12, Gibco) supplemented with 10% fetal bovine serum (FBS, Gibco), 100 IU/mL penicillin (Baomanbio, Shanghai, China), and 100 mg/mL streptomycin (Baomanbio), and then cultured in the incubator at 37 °C in a humidified atmosphere containing 5% CO_2_. Chondrocytes were passaged until a 90% confluence was met. Passage 2 chondrocytes were used for following experiments.

### 4.3. Plasmids Construction and Cell Transfection

FGF-18 fragments containing a miR-195 binding site were amplified and cloned into pmirGLO vector (Promega, Madison, WI, USA) to gain the reporter vector pmiRGLO-FGF-18-wild-type (pmirGLO-FGF-18-wt). The putative binding site of miR-195 in FGF-18 was mutated by using a QuikChange Site-Directed Mutagenesis kit (Agilent, Santa Clara, CA, USA) to synthetize pmirGLO-FGF-18-mutant-type (pmirGLO-FGF-18-mut). The above plasmids were used for the following luciferase reporter assays. Similarly, FGF-18 fragments containing a miR-195 binding site were amplified and cloned into the KpnI and XhoI restriction sites (Promega) of pcDNA3.1 vector to synthetize pcDNA3.1-FGF-18-wild-type (pcDNA3.1-FGF-18-wt), and pcDNA3.1-FGF-18-mutant-type (pcDNA3.1-FGF-18-mut) was also gained by using a QuikChange Site-Directed Mutagenesis kit (Agilent). These two plasmids were used to construct FGF-18 over-expression cell models. For cell transfection, once the chondrocytes were 80% confluent, miR-195 mimics, mimic control, miR-195 inhibitors, inhibitor control (GenePharma, Shanghai, China), and the constructed plasmids were transfected into the chondrocytes with Lipofectamine 2000 (Invitrogen, Carlsbad, CA, USA) according to the manufacturer’s instructions, respectively.

### 4.4. Cell Proliferation Assays

Cell counting Kit-8 (CCK-8) assay and 5-Ethynyl-20-Deoxyuridine (EDU) incorporation assay were used to evaluate the chondrocytes proliferation abilities. For the CCK-8 assay, as previously reported [71], chondrocytes were seeded in 96-well plates (2 × 10^3^) and supplemented with complete growth medium and followed by different transfection 24 h later. At days 1, 2, 3, 4, and 5 after transfection, 10 μL CCK-8 solution was added into each well and incubated for 2 h. Absorbance was measured at 450 nm with a Microplate Autoreader (Bio-Rad, Hercules, CA, USA). Experiments were repeated in triplicate. For EDU incorporation assay, EDU detection kits (Ribobio, Guangzhou, China) were applied and the detailed processes were performed according to the manufacturer’s instructions.

### 4.5. Reverse Transcription and Quantitative Real-Time PCR

The procedure was performed as previously reported [72]. Briefly, total RNAs from joint fluid specimens, chondrocytes, and animal model articular cartilage were isolated by Trizol reagent (Invitrogen) as recommended by the manufacturer’s protocol, respectively. cDNA was reverse transcribed using M-MLV (Invitrogen) containing 5 μg total RNA, then qPCR was performed using SYBR Mixture (CWBIO, Beijing, China). U6 and β-actin were used as an internal control. Primers were synthesized by Sangon Biotech (China) and the sequences were as follows.

**Name****Forward Primer (5′–3′)****Reverse Primer (5′–3′)***FGF-18*CGAGGACGGGGACAAGTATGCAGCTCAGTCTGTCCCTTGG*SOX9*TTCCTCCTCCCGGCATGAGTGCAACTTTGCCAGCTTGCACG*Col2a1*CCCCTGCAGTACATGCGGCTCGACGTCATGCTGTCTCAAG*Aggrecan*TAAACCCGGTGTGAGAACCGCCTGGGTGACAATCCAGTCC*MMP-13*CAGTTGACAGGCTCCGAGAACGTGTGCCAGAAGACCAGAA*GAPDH*GGAATCCACTGGCGTCTTCAGGTTCACGCCCATCACAAAC

### 4.6. Western Blot Analysis

Radio immunoprecipitation assay (RIPA) lysis buffer (Sigma, St. Louis, MO, USA) was used to split cells. Protein samples were subjected to 10% dodecyl sulfate, sodium salt (SDS)-Polyacrylamide gel electrophoresis (SDS-PAGE) and transferred onto a polyvinylidene fluoride (PVDF) membrane, then blocked for 1 h at room temperature. Each membrane was incubated with primary antibodies at 4 °C overnight and then secondary antibodies at room temperature for 1 h the next day. The following antibodies were used: mouse anti-FGF-18 antibody (Absci, Vancouver, WA, USA; dilution rates of 1:1000), mouse anti-FGFR3 antibody (Abcam, Cambridge, UK; dilution rates of 1:1000), mouse anti anti-phospho-FGFR3 antibody (Abcam; dilution rates of 1:1000), mouse anti-ERK1/2 antibody (Abcam, dilution rates of 1:1000), mouse anti-phospho-ERK1/2 (Cell Signaling Technologies, Danvers, MA, USA; dilution rates of 1:1000), and mouse anti-GAPDH antibody (Abcam, concentration of 0.5 µg/mL).

### 4.7. Immunofluorescence Analysis

The procedure was performed as previously reported [73]. Briefly, chondrocytes were seeded onto glass coverslips (0.8 cm × 0.8 cm) and cultured until reaching 50–60% confluence. The medium was abandoned and the coverslips were rinsed twice with phosphate buffer saline (PBS), fixed with 4% paraformaldehyde for 15 min and blocked with 5% bovine serum albumin (BSA) for 1 h at room temperature and incubated with primary antibodies at 4 °C overnight. The next day, after incubation with fluorescent secondary antibodies (Invitrogen) for 1 h and finally incubated with Hoechst 33342 (Cell Signaling Technologies) for 5 min at room temperature, the coverslips were observed under a fluorescent microscope (Leica, Wetzlar, Germany). Images were analyzed using Image-Pro Plus 6.0 software (Media Cybernetics, Rockville, MD, USA).

### 4.8. Dual Luciferase Reporter Assay

HEK-293 cells were seeded in 96-well plates in Dulbecco’s Modified Eagle Media-High Glucose (DMEM-HG, Gibco) supplemented with 10% fetal bovine serum (FBS, Gibco), 100 IU/mL penicillin (Baomanbio), and 100 mg/mL streptomycin (Baomanbio) and cultured for 24 h. miR-195 mimics, miR-195 mimic control, and the constructed reporter plasmids—pmirGLO-FGF-18-wt/pmirGLO-FGF-18-mut—were co-transfected into the cultured HEK-293 cells when they reached about 60–80% confluence. Forty-eight hours after transfection, the luciferase activity was measured using the Dual-Luciferase Reporter Assay System according to the manufacturer’s protocol (Promega).

### 4.9. Establishment of Chronic Cartilage Lesion Models in Rat

We used the anterior cruciate ligament transection (ACLT) method to simulate the chronic cartilage lesion models in rat specimens, as previously reported [74]. In brief, fifteen 8-week-old rats were divided into three groups randomly: sham surgery group (*n* = 5, non-ACLT and intra-articular injection of sterile saline), antagomir NC group (*n* = 5, ACLT and intra-articular injection of antagomir negative control), and miR-195 antagomir group (*n* = 5, ACLT and intra-articular injection of miR-195 antagomir). All rats were operated on using the right knee after sterilization with polyvinyl iodine (TargetMol, Boston, MA, USA). A medial parapatellar incision was made and the patella was dislocated laterally to expose the anterior cruciate ligament (ACL), then ACL was transected by using a #11 surgical blade in antagomir NC group and miR-195 antagomir group, but not in antagomir NC group, respectively. The medial retinaculum was repaired, and the capsule and skin was layer sutured. For the postoperative injection, 10 mg/kg antagomir negative control, 10 mg/kg miR-195 antagomir (Ribobio, Guangzhou, China), and the same volume of sterile saline was injected into the operated knee joint cavities fortnightly in the antagomir NC group, miR-195 antagomir group, and sham surgery group, respectively. After 8 weeks injection, the rats were sacrificed for further detection.

### 4.10. Samples Collection and Immunohistochemistry

The cartilage samples of tibial plateau containing 2 cm thickness of subchondral bones were collected from the sacrificed rats for further immunohistochemistry (IHC) detection. The samples were fixed in 4% paraformaldehyde for 24 h, and then decalcified in 10% ethylene diamine tetraacetic acid (EDTA) (pH 7.3), dehydrated, and paraffin embedded. Slices of 5 µm were taken through the tissue samples at an interval of 50 µm. Safranin O staining was performed by using of Safranin O staining kit (Keygen, Nanjing, China) to evaluate the degree of chronic cartilage lesion according to the manufacturer’s instructions. IHC staining was executed to measure the expression of Col2a1/aggrecan, as previously described [75]. Briefly, slices were incubated with primary antibodies at 4 °C overnight and secondary antibodies at 37 °C for 30 min consecutively, and were subsequently incubated with streptavidin horseradish peroxidase, stained with 3,3-diaminobenzidine, then counterstained with hematoxylin, dehydrated in a graded ethanol series (absolute ethyl alcohol for 3 min, 95% ethanol for 3 min, and 85% ethanol for 3 min), and finally mounted.

### 4.11. Statistical Analysis

All experiments were repeated in triplicate and all data from three independent experiments were expressed as mean ± SD. GraphPad Prism V5.0 (GraphPad Software, Inc., La Jolla, CA, USA) software and SPSS 19.0 statistical software were used for statistical analysis. Correlation between miR-195 and FGF-18 expression was determined by two-tailed Pearson’s correlation analysis. Differences in two groups were analyzed with the Student’s *t*-test or one-way ANOVA. Differences were considered significant if *p* < 0.05 and differences were considered prominently significant if *p* < 0.01.

## 5. Conclusions

In summary, the current study demonstrated that miR-195 was closely involved in the proliferation of chondrocytes and maintenance of chondrogenic phenotype via targeting FGF-18 and its downstream pathway. Decreases in miR-195 promoted chondrocyte proliferation and maintenance of chondrogenic phenotype. Therefore, the present study indicated that the miR-195/FGF-18 pathway might be a new target in cartilage regeneration and repair.
